# The Effect of Preoperative Hydration on Cardiac Surgery-Associated Acute Kidney Injury

**DOI:** 10.21470/1678-9741-2024-0354

**Published:** 2026-05-06

**Authors:** Ayse Zehra Karakoc, Esra Ozcan, Omer Faruk Akardere, Deniz Cevirme, Ahmet Zengin, Hasan Sunar

**Affiliations:** 1 Department of Cardiovascular Surgery, Sehit Prof. Dr. İlhan Varank Sancaktepe Research and Training Hospital, Istanbul, Türkiye; 2 Department of Cardiovascular Surgery, Kartal Kosuyolu Heart Research and Training Hospital, Istanbul, Türkiye; 3 Department of Cardiovascular Surgery, Istınye Unıversity Faculty of Medicine, Istanbul, Türkiye; 4 Department of Cardiovascular Surgery, Fatih Gebze State Hospital, Kocaeli, Türkiye

**Keywords:** Saline Solution, Glomerular Filtration Rate, Hospital Mortality, Creatinine, Control Groups, Cardiac Surgical Procedures, Acute Kidney Injury.

## Abstract

**Objective:**

The aim of this study was to determine the effect of preoperative intravenous saline hydration on postoperative renal functions and the prevention of acute kidney injury subsequent to open-heart surgery.

**Methods:**

Our investigation was designed as a prospective, randomized, and controlled single-center trial. We included 110 patients with basal renal functions that were not disrupted and who were undergoing cardiac surgery from October to December 2020. The first group (control) had fluid restriction for 12 hours prior to surgery (n = 55), and the second group (case) was hydrated with 0.9% normal saline for 12 hours before surgery (n = 55).

**Results:**

In the hydration group, creatinine values dropped below the preoperative values (P = 0.008) and the glomerular filtration rate values rose above the preoperative levels (P = 0.003). The early-term mortality rates were 0% for the hydration group and 5.45% for the control group (n = 3). Besides, in the hydration group, the glomerular filtration rate values on the 30^th^ day and 360^th^ day after surgery increased to levels even higher than those recorded preoperatively.

**Conclusions:**

As a result of much effort, we showed that preoperative hydration can prevent severe cardiac surgery-associated acute kidney injury and related in-hospital mortality.

## INTRODUCTION

**Figure:**
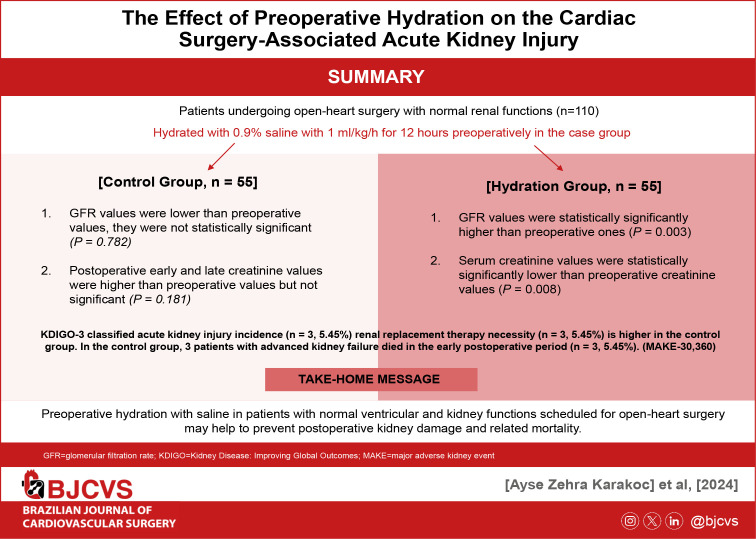

**Table:** 

Abbreviations, Acronyms & Symbols				
ACE-Is	= Angiotensin-converting enzyme inhibitors		IABP	= Intra-aortic balloon pump
AKI	= Acute kidney injury		ICU	= Intensive care unit
AKIN	= Acute Kidney Injury Network		INR	= International Normalized Ratio
ARBs	= Angiotensin II receptor blockers		KDIGO	= Kidney Disease: Improving Global Outcomes
AV ECMO	= Arteriovenous extracorporeal membrane oxygenation		LVEF	= Left ventricular ejection fraction
BMI	= Body mass index		MAKE	= Major adverse kidney event
BSA	= Body surface area		MCS	= Mechanical circulatory support
CABG	= Coronary artery bypass grafting		MI	= Myocardial infarction
CAG	= Coronary angiography		NSAIDs	= Nonsteroidal anti-inflammatory drugs
CKD-EPI	= Chronic Kidney Disease Epidemiology Collaboration		PAD	= Peripheral arterial disease
COPD	= Chronic obstructive pulmonary disease		PCI	= Percutaneous coronary intervention
CRP	= C-reactive protein		PLT	= Platelet
CSA-AKI	= Cardiac surgery-associated acute kidney injury		POP	= Postoperative
CVE	= Cerebrovascular event		Preop	= Preoperative
DM	= Diabetes mellitus		RBC	= Red blood cell
ECMO	= Extracorporeal membrane oxygenation		RHC	= Right heart catheterization
EF GIS	= Ejection fraction = Gastrointestinal system		RIFLE	= Risk, Injury, Failure, Loss of kidney function, and End-stage kidney disease
GFR	= Glomerular filtration rate		RRT	= Renal replacement therapy
HGB	= Hemoglobin		VV ECMO	= Venovenous extracorporeal membrane oxygenation
HT	= Hypertension		WBC	= White blood cell
HTC	= Hematocrit			

Cardiac surgery-associated acute kidney injury (CSA-AKI) is a prevalent and serious complication, with incidence rates ranging from 5% to 42%^[1]^ depending on the clinical setting^[2,3]^. The requirement for renal replacement therapy (RRT) in these cases is approximately 2% to 5%^[4-6]^.

Although CSA-AKI has been defined simply as a rapid deterioration in renal functions and a significant decrease in glomerular filtration rate (GFR) following cardiac surgery, considerations related to the amount and duration of urine output and the presence of oliguria have changed over the years, meaning a complete consensus has not been reached about the definition of this term^[7,8]^.

At present, there are three general classifications for acute kidney injury (AKI). The Risk, Injury, Failure, Loss of kidney function, and End-stage kidney disease (RIFLE) criteria were first introduced in 2004, but shortly after, the Acute Kidney Injury Network (AKIN) introduced a new classification by defining the minimal increase in creatinine in the first four hours postoperatively as stage 1 AKI^[9-11]^. The most widely accepted classification today is the Kidney Disease: Improving Global Outcomes (KDIGO) criteria which synthesizes elements from both RIFLE and AKIN^[12,13]^.

Sutherland et al. suggested three new categories (transient, late onset, and sustained AKI) according to the initiation and duration of changes in creatinine levels and urine output^[10,14]^. Additionally, the Society of Thoracic Surgeons Database has incorporated major adverse kidney events within 30, 90, and 360 days post-surgery (major adverse kidney event [MAKE] 30, 90, and 360) as indicators of early-stage mortality^[14,15]^.

The pathogenesis of CSA-AKI is multifactorial, involving preoperative renal ischemia, reperfusion injury, oxidative stress, inflammation, hemolysis induced by cardiopulmonary bypass, and pigment nephropathy^[2,6]^. It has been predicted that hypovolemia and preoperative fluid restriction could also lead to postoperative AKI, however fluid restriction is still applied in many clinics^[13,16-18]^. This study investigates whether preoperative intravenous hydration, as opposed to the traditional fluid restriction, can effectively reduce the incidence of postoperative CSA-AKI.

## METHODS

The ethical approval of the study was obtained from the Health Sciences University Kartal Kosuyolu High Specialization Training and Research Hospital’s Clinical Research Ethics Committee (approval date: 22/09/2020, and approval number: 2020/8/361), and clinical registry number is ISCRTN10684639.

A hundred and ten patients scheduled for open-heart surgery between October 2020 and December 2020 at Kosuyolu Heart Hospital, with a GFR > 45 ml/min/1.73 m^2^, not undergoing dialysis and without ventricular dysfunction were randomly divided into two groups. The first group was traditionally left with fluid restriction overnight before surgery, while the other 55 patients were subjected to preoperative 12-hour intravenous hydration with 0.9% saline. During this process, their total fluid intake and outputs were monitored to avoid any hypervolemia. Additionally, angiotensin-converting enzyme inhibitors (ACE-Is) and angiotensin II receptor blockers (ARBs) group medications were discontinued preoperatively in our clinic, and our patients in the study group were given alternative antihypertensive agents postoperatively. All patients were informed before the operation and their consents were obtained.

G-power analysis was performed, and 110 patients were determined with a 1:1 randomization for the two groups. For those patients who received intravenous hydration, 0.9% saline solution was used, as it has the closest chloride and electrolyte content to serum values. It was administered as an infusion at a rate of 1cc/kg/hour for 12 hours. The same anesthesia protocols were applied intraoperatively and postoperatively. Blood urea nitrogen, creatinine, and GFR values, as well as blood gas values (pH, lactate, HCO₃, Na^++^, K^+^, etc.) on postoperative days zero, first, and second, were assessed at follow-ups on postoperative days one, two, three, and seven, and at the first month and the first year.

It has been shown that the GFR value, calculated using the Chronic Kidney Disease Epidemiology Collaboration (CKD-EPI) formula, is superior to the Modification of Diet in Renal Disease (or MDRD) in defining stage 2 kidney injury cases, and this has even been mentioned in the KDIGO guidelines^[13,18-20]^. In our study, estimated GFR was calculated using the CKD-EPI method in light of this knowledge and used accordingly.

### Exclusion Criteria

Patients undergoing emergency surgery and aortic dissection, complex aortic surgery patients, congenital heart surgery patients, minimal invasive patients, and redo patients were not included (due to long perfusion and cross-clamping times). Additionally, advanced-stage or end-stage chronic kidney failure patients receiving preoperative RRT (GFR < 45 ml/min/1.73 m^2^) were also excluded.

### Surgical Procedure

All operations were performed according to modern cardiopulmonary bypass principles. Traditional median sternotomy was applied to all patients. Cardiopulmonary bypass was used in all patients, and off-pump surgeries were not included. Moderate hypothermia was applied. Mean perfusion pressure was maintained at 50 - 80 mmHg, and flow rate was kept stable at 2.2 - 2.5 L/min/m^2^, with hematocrit levels maintained at 20 - 25%. As the myocardial protection strategy, all patients received intermittent antegrade and continuous retrograde isothermic and hyperkalemic blood cardioplegia using a combined technique.

### Postoperative Follow-up

All patients were monitored in the same intensive care unit (ICU), following the same anesthesia protocols. Postoperative RRT was initiated in cases of fluid overload resistant to treatment, severe hyperkalemia (plasma K+ > 6.5 mEq/l) or rapidly increasing potassium levels, uremic symptoms such as encephalopathy, pericarditis, or unexplained mental regression, and severe metabolic acidosis (pH < 7.1)^[21]^.

### Statistical Analysis

G-power analysis was used to determine the total sample size and with an effect size of 0.48 (alpha error probability = 0.05) and a power value of 0.80, the total required sample size was found to be 110 (at least 55 patients for each group). IBM SPSS Statistics for Windows, version 23 (IBM Corp. Armonk, N.Y., USA) software package was used for data analysis. Descriptive statistics were presented as mean ± standard deviation and median, minimum-maximum for continuous variables, and frequency and (%) for categorical variables.

As a result of the analyses, the demographic data (sex, age, etc.) were presented as mean ± standard deviation and median, minimum-maximum for continuous variables, and frequency and (%) for categorical variables.

The Kolmogorov-Smirnov test was applied as the normality distribution test. For comparison of variables with non-normal distribution, the Mann-Whitney U test was used to examine whether there was a difference between the groups since the groups were independent. The Wilcoxon test was used to measure the effectiveness of measurement methods, while the Chi-square test and Fisher’s exact test value were used to test whether there was a difference between categorical variables. Repeated measurements within the same group were tested using the repeated analysis of variance test. A statistical significance level of *P* ≤ 0.05 was accepted. Early postoperative (one month) and late postoperative (one year) survival analyses were evaluated and compared using life-table analysis.

## RESULTS

### Examination Results of Preoperative Baseline Demographic, Clinical, and Biochemical Characteristics for Both Groups

The demographic data of both groups are given in Table 1 with distributions and comparisons between the two groups indicated as *P*-values. No significant difference was observed between the preoperative demographic data of both groups. No significant differences were observed between the preoperative demographic data, laboratory values, and cardiac functions for both groups (Tables 2 and 3).

**Table 1:** Distribution and comparison of preoperative demographic data and comorbidities between case and control groups.

Variable		Control group	Case group	*P*-value
		(n = 55)	(n = 55)	
Age		59.38 ± 8.332 (39 - 78)	57.54 ± 9.441 (40 - 76)	0.284
Sex	Female	46 (83.6%)	41 (74.6%)	
	Male	9 (16.4%)	14 (25.4%)	
BSA		1,9558 ± 1,8802 (1.54 - 2.47)	2,6786 ± 5,10813 (1.61 - 40.2)	0.090
BMI		28,7909 ± 4,62154 (22 - 43)	29,8193 ± 6,04266 (20.8 - 44.6)	0.078
DM		34 (61.8%)	39 (70.9%)	0.423
HT		34 (61.8%)	40 (72.7%)	0.223
PAD		7 (12.7%)	4 (7.3%)	0.34
COPD		10 (18.2%)	13 (23.6%)	0.165
Cerebrovascular event		4 (7.3%)	5 (9.1%)	0.50
GIS diseases		5 (9.1%)	1 (1.8%)	0.206
AKI history		1 (1.8%)	0	1
Thyroid dysfunction		3 (5.5%)	5 (9.1%)	0.716

AKI=acute kidney injury; BSA=body surface area; BMI=body mass index; COPD=chronic obstructive pulmonary disease; DM=diabetes mellitus; GIS=gastrointestinal system; HT=hypertension; PAD=peripheral arterial disease

**Table 2:** Comparison of preoperative cardiac functions and need for mechanical support between the two groups.

Variable	Control group	Case group	*P*-value
	(n = 55)	(n = 55)	
Arrhythmia	3 (5.5%)	2 (3.6%)	1
< 5 days before RHC/CAG	10 (18.2%)	9 (16.1%)	0.80
Preoperative IABP	1 (1.81%)	1 (1.81%)	1
Preoperative MCS (ECMO)	0	0	1
EF (%)	59.81 ± 7.32 (45-65)	59.55 ± 6.69 (45 - 65)	0.700

CAG=coronary angiography; ECMO=extracorporeal membrane oxygenation; EF=ejection fraction; IABP=intra-aortic balloon pump; MCS=mechanical circulatory support; RHC=right heart catheterization

**Table 3:** Distribution and comparison of preoperative baseline laboratory values between case and control groups.

Variables	Case group	Control group	*P*-value
	(n = 55)	(n = 55)	
Preoperative WBC	8324,81 ± 2522,16 (4400 - 15900)	8042,65 ± 2719,54 (4900 - 12700)	0.984
Preoperative HTC	40.68 ± 5.98 (25.9 - 53.6)	40.29 ± 4.0 (29.3 - 49.3)	0.201
Preoperative HGB	13.58 ± 2.14 (8.2 - 17.7)	13.55 ± 1.38(10.1 - 16.7)	0.429
Preoperative RBC	4.67 ± 0.60 (2.93 - 5.89)	4.65 ± 0.50 (3.37 - 5.90)	0.397
Preoperative PLT	245772,25 ± 64626,07 (146000 - 415000)	236594,49 ± 87613,62 (139000 - 416000)	0.953
Preoperative INR	1.04 ± 0.08 (0.83 - 1.27)	1.06 ± 0.10 (0.89 - 1.43)	0.418
Preoperative glucose	153.82 ± 66.6 (80 - 342)	159.34 ± 74.3 (81 - 383)	0.894
Preoperative urea	35.7925 ± 10.85 (19 - 65)	37.06 ± 14.5 (19 - 123)	0.988
Preoperative creatinine	0.90 ± 0.24 (0.47 - 1.67)	0.83 ± 0.17 (0.51 - 1.25)	0.132
Preoperative GFR	87.03 ± 19.9 (46 - 124)	91.7 ± 15.22 (49 - 117)	0.168
Preoperative potassium	4.39 ± 0.44 (3.5 - 5.34)	4.34 ± 0.35 (3.62 - 5.21)	0.416
Preoperative sodium	138.2 ± 2.69 (133 - 144)	137.83 ± 2.62 (131 - 145)	0.666
Preoperative albumin	41.66 ± 4.66 (28 - 51)	40.67 ± 3.64 (32 - 48)	0.17
Preoperative CRP	11.90 ± 18.34 (0.93 - 85)	14.70 ± 26.04 (0.53 - 148)	0.379
Preoperative troponin	0.17 ± 0.54 (0.001 - 3.72)	0.16 ± 0.44 (0.001 - 0.44)	0.593

CRP=C-reactive protein; GFR=glomerular filtration rate; HGB=hemoglobin; HTC=hematocrit; INR=International Normalized Ratio; PLT=platelet; RBC=red blood cell; WBC=white blood cell

### Evaluation the Results and Comparison of Operation-Related and Intraoperative Data

No significant difference was found between the case and control groups regarding operation types (isolated coronary artery bypass grafting, isolated valve, and combined surgery), cross-clamping times, total perfusion or cardiopulmonary bypass times, and total operation times (*P* > 0.05). As shown in Table 4, intraoperative total urine output and total fluid balance distribution and amounts also showed no significant difference.

**Table 4:** Comparison of intraoperative data and surgical types between the two groups.

Variables	Case group	Control group	*P*-value
	(n = 55)	(n = 55)	
Isolated CABG	52 (94.5%)	51 (92.7%)	0.7
Isolated valve	2 (3.63%)	3 (5.45%)	1
CABG + valve	1 (1.8%)	1 (1.8%)	1
Cross-clamping time	67.07 ± 29.2 (17 - 153)	68.67 ± 28.3 (21 - 166)	0.77
Total perfusion time	111.5 ± 40.67 (50 - 225)	114.85 ± 34.7 (52 - 206)	0.643
Total operation time	272.57 ± 62.8 (134 - 425)	265.7 ± 56.63 (138 - 395)	0.546
Total urine output	1324 ± 516.4 (600 - 3200)	1292 ± 485.7 (480 - 2170)	0.735
Total fluid balance	1255.36 ± 856.8 (-320-(+ 3350))	1058.36 ± 690.15 (-20-(+ 3200))	0.233

CABG=coronary artery bypass grafting

### Postoperative Primary and Secondary Outcomes

During ICU stay, whether there was a difference in terms of arterial blood gas values on postoperative day zero (first 24 hours) for lactate, pH, potassium, sodium, chloride, and bicarbonate variables was evaluated using the Mann-Whitney U test (Table 5).

**Table 5:** Comparison of blood gas values in the first 24 hours.

Variables	Case group	Control group	*P*-value
	(n = 55)	(n = 55)	
Lactate (mmol/L)	3.02 ± 1.35 (1.2 - 6)	3.89 ± 2.05 (0.7 - 13)	0.016
pH	7.39 ± 0.37 (7.35 - 7.48)	7.36 ± 0.19 (7 - 7.51)	0.389
Potassium level (mmol/L)	4.1 ± 0.49 (3.1 - 5)	4.12 ± 0.46 (3.1 - 5.2)	0.981
Sodium level (mmol/L)	140.45 ± 2.92 (132 - 146)	140.76 ± 3.89 (131 - 153)	0.628
Chloride level (mmol/L)	114.18 ± 2.9 (105 - 120)	114.58 ± 3.3 (108 - 123)	0.209
Bicarbonate level (mmol/L)	22.03 ± 1.46 (18 - 25.2)	21.52 ± 1.38 (18.3 - 24.3)	0.632

According to the test results, based on the hydration status, the average lactate values for the hydrated case group are statistically significantly lower than the average values for the group not receiving hydration (*P* = 0.016). No statistically significant difference was detected for the other variables.

Additionally, no significant difference was observed between the groups in terms of any miscellaneous complications that developed within the first 48 hours postoperatively, according to the Chi-square test results (Table 6).

**Table 6:** Comparison of complications and the need for cardiac and renal medical or mechanical support between the hydration group and the control group in the first 24 postoperative hours.

Variables	Case group	Control group	*P*-value
	(n = 55)	(n = 55)	
Arrhythmia	7 (12.7%)	11 (20%)	0.303
Revision for bleeding	0 (0%)	2 (3.6%)	0.248
Renal dose dopamine application	12 (21.8%)	12 (21.8%)	1
Furosemide application	13 (23.6%)	16 (29.1%)	0.516
Inotrope requirement	8 (14.5%)	13 (23.6%)	0.225
MCS (IABP)	2 (3.6%)	3 (5.5%)	0.647
MCS (AV ECMO)	0 (0%)	1 (1.8%)	1
MCS (VV ECMO)	1 (1.8%)	0 (0%)	1

AV ECMO=arteriovenous extracorporeal membrane oxygenation; IABP=intra-aortic balloon pump; MCS=mechanic circulatory support; VV ECMO=venovenous extracorporeal membrane oxygenation

For the participants in the study who were hydrated and the control group, preoperative, postoperative day one, day two, one-week, and one-month GFR, creatinine, and urea values, as well as the replacement fluids and blood products, were examined for both groups. Although the hydrated group generally showed a higher urine output in the early postoperative period compared to the control group, the difference was not found to be significant (Table 7).

**Table 7:** Comparison of urine outputs (cc/kg/h) and total urine output in the first 24 hours between the two groups.

Urine output	Control group	Case group	*P*-value
	(n = 55)	(n = 55)	
0 - 12 h (ml/kg/h)	1.27 ± 1.03	1.34 ± 1.07 (0.27 - 6)	0.337
12 - 24 h (ml/kg/h)	1.05 ± 0.69	1.15 ± 1.02 (0.21 - 6.25)	0.763
24 - 36 h (ml/kg/h)	0.99 ± 0.46	1.16 ± 0.59 (0.28 - 3.5)	0.269
36 - 48 h (ml/kg/h)	1.05 ± 0.48 (0 - 3.8)	1.17 ± 0.65 (0.25 - 3.57)	0.443
Total 24 hours (ml)	3033 ± 988 (1540 - 6700)	2865 ± 772 (1700 - 5500)	0.414

The test result showed a statistically significant difference between postoperative day seven creatinine and preoperative creatinine values, as well as postoperative day seven GFR and preoperative GFR values in the hydrated group. Postoperative day seven creatinine values were statistically significantly lower than preoperative creatinine values (*P* = 0.008) and postoperative day seven GFR values were statistically significantly higher than preoperative GFR values (*P* = 0.003) (Figure 1A and 1B). Additionally, the GFR values in the hydrated group at postoperative one year (MAKE-360) were even higher than preoperative values, and the increase in GFR values was observed to be significant (*P* = 0.025) (Figure 1B). Table 8 and Table 9 show comparisons between both groups with mean preoperative, postoperative day one, day seven, one-month, and one-year creatinine values and mean GFR values.

**Table 8:** Comparison of preoperative and postoperative serum creatinine values in two groups.

	Case group	Control group	*P*-value
	(n = 55)	(n = 55)	
Preoperative serum creatinine (mg/dl)	0.91 ± 0.25 (0.47 - 1.67)	0.83 ± 0.17 (0.51 - 1.25)	0.130
Postoperative 1^st^ day serum creatinine	0.99 ± 0.31 (0.50 - 1.91)	0.87 ± 0.25 (0.51 - 1.52)	0.060
Postoperative 7^th^ day serum creatinine	0.88 ± 0.35 (0.34 - 2.62)	0.87 ± 0.49 (0.48 - 4)	0.654
Postoperative 30^th^ day serum creatinine	0.92 ± 0.34 (0.42 - 2.67)	0.84 ± 0.15 (0.58 - 1.35)	0.113
Postoperative 360^th^ day serum creatinine	0.90 ± 0.31 (0.4 - 2.61)	0.87 ± 0.16 (0.60 - 1.41)	0.953

**Table 9:** Comparison of preoperative and postoperative glomerular filtration rate (GFR) values (Chronic Kidney Disease Epidemiology Collaboration) in patients.

	Case group	Control group	*P*-value
	(n = 55)	(n = 55)	
Preoperative GFR (ml/min/1.73 m^2^)	86.82 ± 20.01 (46 - 124)	91.18 ± 15.16 (49 - 117)	0.143
Postoperative 1^st^ day	81.35 ± 22.03 (35 - 118)	88.5 ± 19.4 (47 - 126)	0.062
Postoperative 7^th^ day	92.52 ± 22.27 (24 - 138)	90.6 ± 22.3 (18 - 128)	0.876
Postoperative 30^th^ day	87.70 ± 19.70 (23 - 129)	87.2 ± 24.74 (40 - 119)	0.439
Postoperative 360^th^ day	91.17 ± 18.94 (39 - 122)	87.42 ± 25.19 (43 -122)	0.673

**Fig. 1A:**
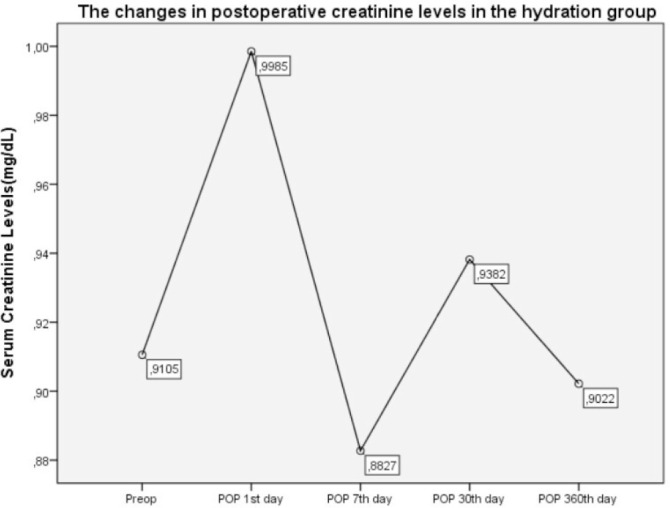
Changes in postoperative (POP) serum creatinine levels in the hydration group. Preop=preoperative.

**Fig. 1B:**
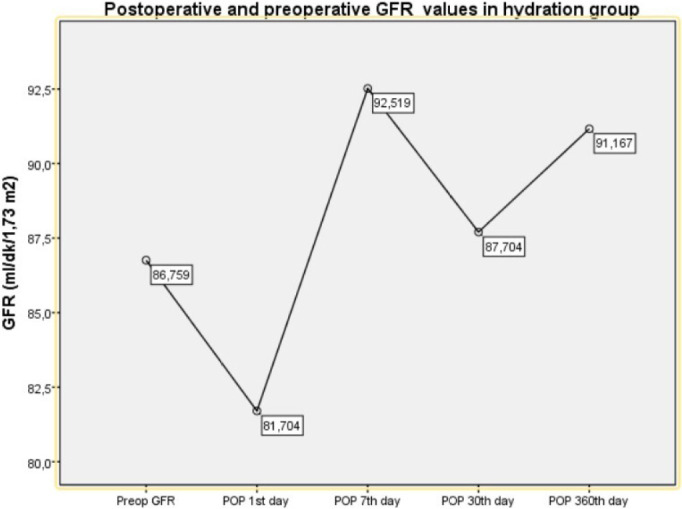
Changes in postoperative (POP) glomerular filtration rate (GFR) values in the hydration group. Preop=preoperative.

In the control group, postoperative early and late creatinine values were higher than preoperative values. The increase in creatinine values was only significantly higher between postoperative one month and one year (*P* = 0.017). Although GFR values were lower than preoperative values, they were not statistically significant (*P* = 0.782) (Figure 2A & 2B).

**Fig. 2A:**
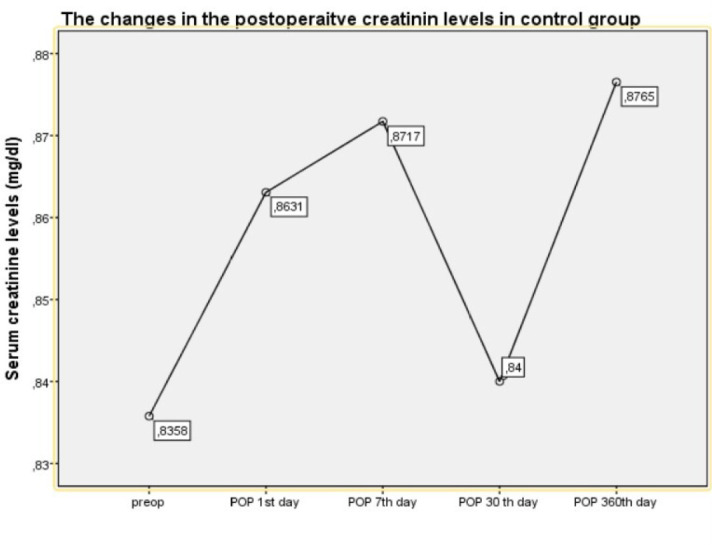
Changes in postoperative (POP) serum creatinine levels in the control group. Preop=preoperative.

**Fig. 2B:**
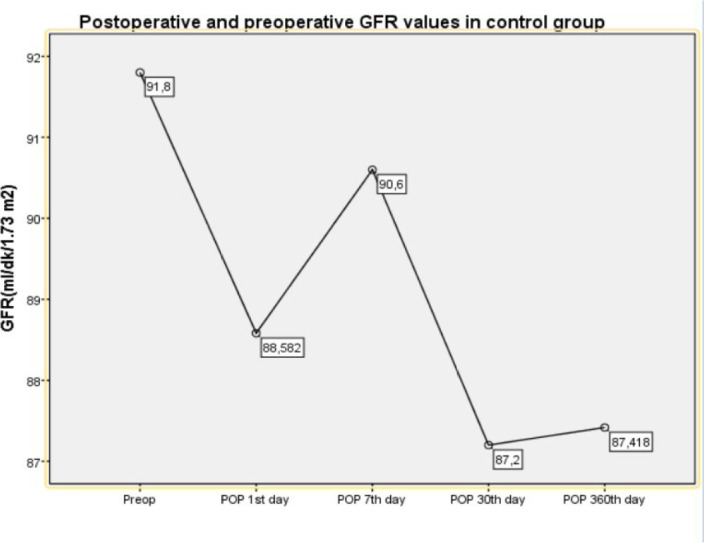
Changes in postoperative (POP) glomerular filtration rate (GFR) values in the control group. Preop=preoperative.

Patients who developed AKI from both groups were categorized as KDIGO 1, 2, and 3, and statistical evaluations were performed (Table 10). No KDIGO-3 advanced-stage AKI was not detected in any patient in the hydrated group. In the control group, however, KDIGO-3 AKI was observed in three patients (n = 3, 5.5%); all three of them experienced adverse outcomes during early ICU stay. Besides, advanced-stage AKI was not seen in any patients in the hydrated group according to all three classifications (KDIGO-3, AKIN-3, and RIFLE-F).

**Table 10:** Comparison of patients' classification according to the three acute kidney injury (AKI) criteria and Sutherland classification.

AKI Classifications	Case group	Control group	*P*-value
	(n = 55)	(n = 55)	
RIFLE-Risk	5 (9.1%)	10 (18.2%)	0.165
RIFLE-Injury	2 (3.6%)	0	0.495
RIFLE-Failure	0	3 (5.5%)	0.243
AKIN-1	6 (10.9%)	10 (18.2%)	0.279
AKIN-2	2 (3.6%)	0	0.248
AKIN-3	0 (0%)	2 (3.6%)	0.243
KDIGO-1	6 (10.9%)	11 (20%)	0.187
KDIGO-2	2 (3.6%)	0	0.248
KDIGO-3	0	3 (5.45%)	0.243
Transient-AKI	6(10.9%)	4(7.3%)	0.507
Sustained-AKI	2(3.6%)	5(9.1%)	0.241
Late-AKI	0	4 (7.3%)	0.042

AKI=acute kidney injury; AKIN=Acute Kidney Injury Network; KDIGO=Kidney Disease: Improving Global Outcomes; RIFLE=Risk, Injury, Failure, Loss of kidney function, and End-stage kidney disease

In the hydrated group, no temporary renal replacement needs were observed, and one patient required permanent hemodialysis during the final stage of a prolonged intensive care stay due to sepsis, resulting in a late-term complication (n = 1, 1.8%). On the other hand, amongst the control group, a total of three patients needed RRT, with two of them requiring permanent RRT (n = 2, 3.6%) and one receiving temporary RRT (n = 1, 1.8%).

Regarding 30-day and 360-day major adverse kidney-related events comorbidity and mortality data, defined as MAKE-30 and MAKE-360, GFR values, RRT requirements, and mortality data were evaluated for both groups at first month and first year. In the survival analysis conducted using the life-table survival analysis method, early survival (MAKE-30) was observed to be 94.54% in the control group, while it was 100% in the hydrated group. In the hydrated group, only one patient died on the 40th day due to sepsis associated with a long intensive care stay (n = 1, 1.8%) and this patient hasn’t had advanced kidney failure. In the control group, all three patients who were lost in the early period were found to have advanced kidney failure and were classified as KDIGO-3 (n = 3, 5.45%). One-year survival was analyzed as 98.18% in the hydrated group and 94.54% in the control group and demonstrated using life-table analysis (Figure 3A and 3B). The all-over results were emphasized as a central image in the Supplemental Figure.

**Fig. 3A:**
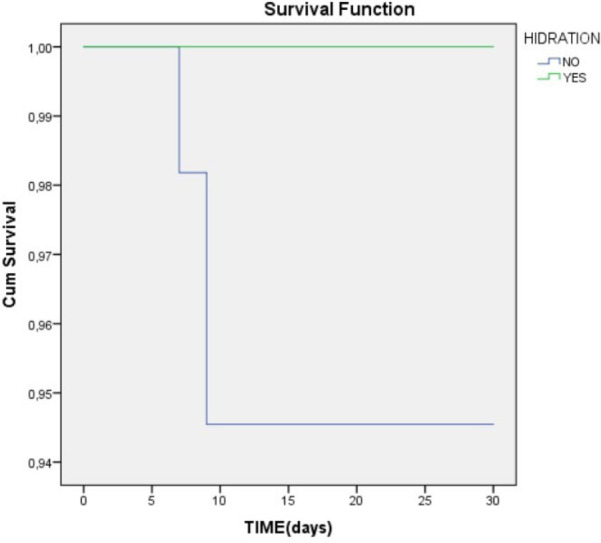
Early-term survival analysis for two groups (MAKE-30). MAKE=major adverse kidney event.

**Fig. 3B:**
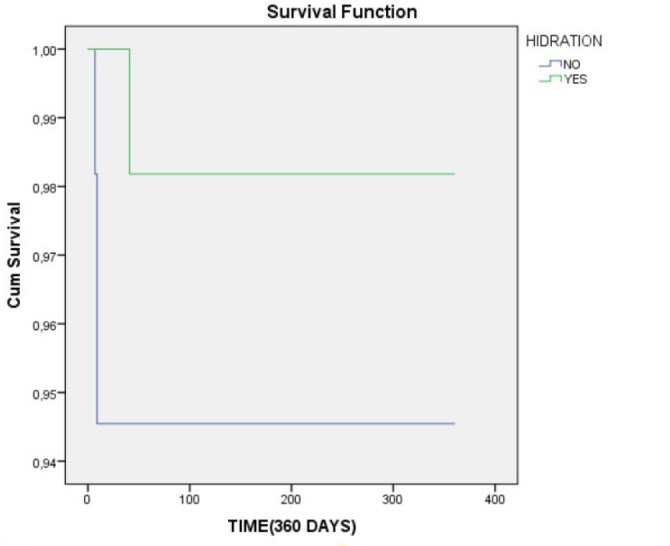
One-year (360 days) survival analysis for two groups (MAKE-360). MAKE=major adverse kidney event.

### Postoperative Secondary Endpoints

The Mann-Whitney U test was used to evaluate the differences between the two groups in terms of total drainage, ICU stay, total hospital stay, and extubation times. The total hospital stay was found to be significantly lower in the hydration group (*P* = 0.049). Additionally, total drainage and extubation times were also lower in the hydration group, but they were not statistically significant (Table 11).

**Table 11:** Comparison of hospital stay, intensive care unit (ICU) stay, total drainage, and extubation durations from postoperative major comorbidity determinant data between the two groups.

Variables	Case group	Control group	*P*-value
	(n = 55)	(n = 55)	
Extubation time (hour)	16.57 ± 43.87 (1 - 336)	16.79 ± 28.15 (6 - 168)	0.809
Total drainage (ml)	664.2 ± 384.08 (50 - 2100)	791.82 ± 560.4 (250 - 370)	0.274
ICU stay (days)	3.18 ± 4.53 (1 - 30)	2.71 ± 2.19 (1 - 12)	0.526
Total hospital stay (days)	8.54 ± 5.97 (4 - 42)	12.78 ± 6.38 (6 - 44)	0.049

No significant difference was observed between the two groups in terms of non-renal complications that developed in the early postoperative period (Table 12). Although no significant difference was found between the groups in terms of preoperative and postoperative one-week and one-year left ventricular ejection fraction (LVEF) averages (Table 13), when preoperative and postoperative values were compared separately within the same group’, no significant difference was observed in the hydration group (*P* = 0.125). However, in the control group, a statistically significant difference was found between postoperative seventh day ejection fraction (EF) and preoperative EF (*P* = 0.011) values, with postoperative EF values being significantly lower than preoperative EF values. Postoperative one-year LVEF values were also found to be significantly lower compared to preoperative values (*P* = 0.048).

**Table 12:** Postoperative miscellaneous complications.

Complications	Case group	Control group	*P*-value
	(n = 55)	(n = 55)	
Arrhythmia	12 (21.8%)	20 (36.4%)	0.303
Pulmonary complications	10 (18.2%)	6 (10.9%)	0.279
Revision for bleeding	2 (3.6%)	3 (5.5%)	1
Revision for tamponade	2 (3.6%)	1 (1.8%)	1
Revision for sternal dehiscence	3 (5.5%)	3 (5.5%)	1
Preoperative MI rates (PCI)	0 (0%)	2 (3.6%)	0.495
Preoperative MI rates (CABG)	1 (1.8%)	1 (1.8%)	1
CVE (ischemia, hemorrhagic)	2 (3.6%)	4 (7.2%)	1
Systemic infection	2 (3.6%)	2 (3.6%)	1

CABG=coronary artery bypass grafting; CVE=cerebrovascular event; MI=myocardial infarction; PCI=percutaneous coronary intervention

**Table 13:** Comparison of preoperative and postoperative one-week and one-year ejection fraction values in both groups.

LVEF values	Case group	Control group	*P*-values
	(n = 55)	(n = 55)	
Preoperative LVEF (%)	59.64 ± 6.79 (45 - 65)	59.81 ± 7.32 (45 - 65)	0.93
Postoperative 7^th^ day LVEF (%)	59.18 ± 5.59 (40 - 65)	56.54 ± 9.12 (30 - 65)	0.26
Postoperative 1^st^ year LVEF (%)	58.98 ± 6.47 (40 - 60)	56.41 ± 15.03 (25 - 60)	0.67

LVEF=left ventricular ejection fraction

## DISCUSSION

AKI is the most common complication subsequent to open-heart surgery (5 - 42%)^[1]^. Although reversibility in most patients, it is known from years of clinical studies to be associated with prolonged ICU stay, myocardial depression, renal deterioration, RRT, and ultimately increased in-hospital and out-of-hospital mortality^[22]^. In our study, we aimed to show the effects of preoperatively maintaining intravascular volume, which is known to be significant for AKI, on kidney functions, additional complications, and ultimately mortality in the early postoperative period (one month) and at one-year follow-up. For this purpose, we used a 0.9% saline solution, which is commonly used in clinics and easily accessible, and does not lead to myocardial depression in patients.

There were no significant differences in preoperative demographic data (such as age, sex, body surface area, body mass index), preoperative laboratory values, or additional comorbidities between the two groups. Postoperatively, close intensive care monitoring and blood gas measurements, hourly urine output, and central venous pressure tracking were performed on the first 24 hours and in the case group; the postoperative lactate value, which we consider an indirect indicator of tissue perfusion and oxygenation in the early postoperative period, was seen to be significantly lower in the case group than in the control group (*P* = 0.016). Postoperative hyperlactatemia is one of the most important indicators of poor prognosis in intensive care patients^[23]^. It has been shown in various studies that it can be directly related to acute respiratory distress syndrome, cardiogenic shock, and mortality, in addition to renal dysfunction^[24,25]^. At this point, the low lactate values seen in the early postoperative period in our case group were evaluated as a good prognosis indicator.

Detailed evaluation results for renal functions, which are the main starting point of our study, have been provided, and patients developing AKI in both groups have been classified according to the KDIGO and Sutherland AKI classification^[10]^. It was observed that KDIGO-3 AKI were 100% associated with mortality, and all of these patients were from the non-hydrated control group. KDIGO-3 kidney injury was not seen in any patient in the case group. The occurrence of late-onset AKI according to Sutherland classification was significantly higher in the control group (*P* = 0.043). In addition to these striking results, although an increase in creatinine was observed temporarily in the first 24 hours and a decrease in GFR could be seen in both the control and hydration groups, these values significantly decreased even below preoperative creatinine levels in the hydrated (case) group in the first week (*P* = 0.008), and the GFR value significantly increased above the preoperative values (*P* = 0.003) in first week and in the annual health control (*P* = 0.025). In terms of the need for renal replacement, no significant difference was observed between the two groups in the early postoperative period and the one-month period (*P* > 0.05).

In our study, evaluations could be made for MAKE-30 and MAKE-360. Results for the early 30-day period and one-year results could be given. Mortality was detected lower in the case group.

To the best of our knowledge, our study is the only prospective randomized clinical study in the literature so far on the effect of preoperative hydration on CSA-AKI. The effect of preoperative hydration on postoperative AKI in abdominal surgery had previously been published by Serrano et al. in 2016, but no significant difference could be demonstrated^[26]^. In only one another study, patients with preoperative chronic kidney disease who were to undergo open-heart surgery were started on hydration and postoperative kidney injury evaluation was manifested. However, because the case group consisted of 30 and the control group of 15 patients, and off-pump surgery patients were also included, it was thought that there could be some severe limitations in examining the sole effect of hydration^[27]^.

Recent studies have indicated that, in addition to clinical criteria, certain novel biomarkers - such as urinary α-1 microglobulin, N-acetyl-β-glucosaminidase, glutathione transferase, and serum and urinary neutrophil gelatinase-associated lipocalin - may be useful in identifying CSA-AKI^[28]^. Moreover, some urinary biomarkers, including interleukin-18 and kidney injury molecule-1, have been observed to not only predict subclinical AKI but also hold prognostic value for three-year mortality following cardiac surgery^[29]^.

The Food and Drug Administration has recently approved the use of urinary tissue inhibitor of metalloproteinases-2 and insulin-like growth factor binding protein-7 biomarkers to aid in the early prediction of moderate-to-severe AKI progression within the first 12 hours postoperatively^[30]^. However, their cost and widespread availability remain below expected levels. While some centers incorporate biomarkers into clinical practice, their high-cost limits broader adoption. As a result, classifications based on serum creatinine levels and urine output continue to serve as the cornerstone for diagnosing CSA-AKI. In further studies, the biomarkers may be used for CSA-AKI diagnosis and determining the long-term follow-up.

Delaying elective surgeries for a few days to discontinue preoperative nephrotoxic agents and consider the effects of contrast media is one of the initial strategies for preventing reversible AKI. The primary nephrotoxic agents include NSAIDs, radiocontrast agents, ACE-Is, and ARBs. In our clinic, we refrain from using ACE-Is, ARBs, and nonsteroidal anti-inflammatory drugs (NSAIDs) for the first 48 - 72 hours postoperatively until hemodynamic stability is achieved and the absence of AKI is confirmed.

Randomized controlled trials have suggested that aspirin and statin therapy should be continued preoperatively^[31,32]^. Additionally, meta-analyses have failed to demonstrate the effectiveness of N-acetylcysteine in improving renal function through its anti-inflammatory and antioxidant properties^[33]^. Consequently, the Acute Disease Quality Initiative guidelines have recommended its removal from routine clinical practice. According to the results of our study, preoperative hydration has been shown to be effective in renoprotection. Therefore, in addition to the aforementioned measures, its routine implementation is recommended.

To the best of our knowledge, our study is the first prospective, randomized clinical trial to assess the isolated effect of exclusive preoperative hydration on CSA-AKI. Lim et al. conducted a retrospective cohort study comparing high (> 1 L) *vs.* low (≤ 1 L) volumes of saline infusion during the first 48 hours postoperatively and found no significant association between infusion volume and AKI incidence, RRT, or mortality^[34]^.

Furthermore, Cardinale et al. pointed out that existing research had largely confined saline administration to intraand postoperative periods and recommended rigorous evaluation of preoperative fluid loading to reduce the incidence of and lessen the severity of CSA-AKI^[35]^.

### Limitations

This study was conducted at a single center, which may restrict the generalizability of the findings. Moreover, renal outcomes were assessed using serum creatinine and estimated GFR without the inclusion of novel biomarkers, which might have limited the detection of subclinical kidney injury. Future studies incorporating novel biomarkers may provide a more comprehensive understanding of the renoprotective effects of preoperative hydration. Additionally, the sample size was powered for the primary renal endpoints and was not designed to detect statistically significant differences in mortality; the observed mortality difference should therefore be interpreted as descriptive only. Finally, the study population was relatively homogeneous, with the majority of patients undergoing isolated CABG, and several high-risk subgroups were excluded by design, which limits the applicability of the findings to more complex surgical cases.

## CONCLUSION

In conclusion, it has been observed that preoperative hydration with saline in patients with normal ventricular and kidney functions scheduled for open-heart surgery may help to prevent postoperative kidney damage and related mortality. In the hydrated group, postoperative creatinine values dropped below preoperative levels, and GFR values rose above preoperative levels, with the GFR benefit sustained at one-year follow-up. No advanced-stage AKI was observed in any patient in the hydration group, and late-onset AKI was significantly more frequent in the control group. Early-term and one-year mortality were both lower in the hydration group, and total hospital stay was significantly shorter compared to the control group. Additionally, postoperative lactate values, which serve as an indirect indicator of tissue perfusion, were lower in the hydration group in the early postoperative period, suggesting better end-organ perfusion. It is believed that the widespread implementation of this simple and cost-effective practice in clinical settings could also reduce kidney injury and related deaths often encountered in the postoperative intensive care duration. Future prospective multicenter trials are needed to further validate these findings across broader patient populations and more complex surgical cases.

## Data Availability

The authors declare that all data for this research were collected prospectively and recorded by the principal investigator using a customized Excel form, which is securely stored in the corresponding author's personal archive. While the entire dataset is not publicly accessible to protect individuals' privacy, a subset of the data (excluding patients' names and identities) will be shared on reasonable request to the corresponding author.

## Artificial Intelligence Usage

The authors declare that no artificial intelligence tool was used in the preparation of this article.

## Clinical Perspective

We suggested hydration prior to cardiac surgery to prevent renal injury instead of the traditional way.

Hydration before cardiac surgery can decrease postoperative acute kidney injury and midterm mortality.
